# Nanofiltration and Tight Ultrafiltration Membranes for the Recovery of Polyphenols from Agro-Food By-Products

**DOI:** 10.3390/ijms19020351

**Published:** 2018-01-24

**Authors:** Alfredo Cassano, Carmela Conidi, René Ruby-Figueroa, Roberto Castro-Muñoz

**Affiliations:** 1 Institute on Membrane Technology, ITM-CNR, c/o University of Calabria, via Pietro Bucci, 17/C, 87036 Rende (CS), Italy; c.conidi@itm.cnr.it; 2 Programa Institucional de Fomento a la Investigación, Desarrollo e Innovación, Universidad Tecnológica Metropolitana, Ignacio Valdivieso 2409, San Joaquín, Santiago 8940577, Chile; rruby@utem.cl; 3 Department of Inorganic Technology, University of Chemistry and Technology Prague Technická 5, 166 28 Prague 6, Czech Republic; castrom@vscht.cz

**Keywords:** polyphenols, biologically active compounds, ultrafiltration, nanofiltration, agro-food by-products

## Abstract

Pressure-driven membrane-based technologies represent a valid approach to reduce the environmental pollution of several agro-food by-products. Recently, in relation to the major interest for natural compounds with biological activities, their use has been also addressed to the recovery, separation and fractionation of phenolic compounds from such by-products. In particular, tight ultrafiltration (UF) and nanolfiltration (NF) membranes have been recognized for their capability to recover phenolic compounds from several types of agro-food by-products. The separation capability of these membranes, as well as their productivity, depends on multiple factors such as membrane material, molecular weight cut-off (MWCO) and operating conditions (e.g., pressure, temperature, feed flow rate, volume reduction factor, etc.). This paper aims at providing a critical overview of the influence of these parameters on the recovery of phenolic compounds from agro-food by-products by using tight UF and NF membranes. The literature data are analyzed and discussed in relation to separation processes, molecule properties, membrane characteristics and other phenomena occurring in the process. Current extraction methodologies of phenolic compounds from raw materials are also introduced in order to drive the implementation of integrated systems for the production of actractive phenolic formulations of potential interest as food antioxidants.

## 1. Introduction

Today, the recovery of biologically active compounds from natural sources has attracted great attention due to their potential uses such as ingredients in foodstuff, pharmaceutics, and cosmetic formulations. Among these compounds, polyphenols (PPs), secondary metabolites ubiquitously distributed in all higher plants, have been recently the most searched ones [1]. PPs represent a wide variety of compounds, which are classified in several classes, such as hydroxycinnamic acids, hydroxybenzoic acids, anthocyanins, flavonols, proanthocyanins, flavonols, flavanones, isoflavones, flavones, stilbenes and lignans [2,3,4]. More than 8000 phenolic structures are currently known, and among them, over 4000 flavonoids have been identified [5].

**Figure 1:**
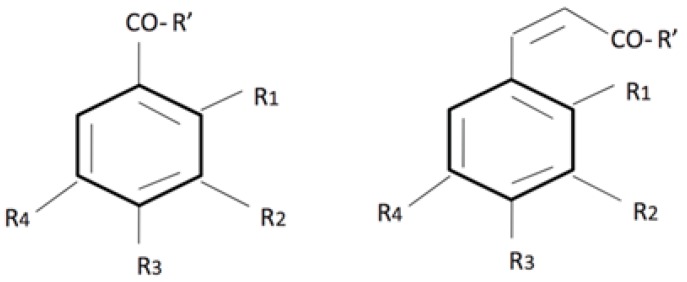
Molecular structure of benzoic and cinnamic acid derivatives.

These plant metabolites are characterized by the presence of several phenol groups (aromatic rings with hydroxyls), which are derived from l-phenylalanine or tyrosine. Typically, all these compounds present the basic chemical structure based on cinnamic acids (C6–C3) and benzoic acids (C6–C1) (Figure 1).

Over the course of the last decades, phenolic compounds have been deeply studied for their great protective activities as antioxidants, free radical scavengers, and metal chelators, as well as to the ability to reduce and inhibit different types of enzymes (telomerase, lipoxygenase, cycloxygenase) [1,6]. They also contribute to the prevention of several types of human diseases, such as cardiovascular disease, cancer, osteoporosis, diabetes, and neurodegenerative diseases. In particular, some phenolic compounds (e.g., flavan-3-ol; flavonol, tannin, neolignan) have demonstrated specific health protective effects such as antimicrobial activity (against virus, bacteria, and fungi) [1] and oral health [4]. Basically, these health-protective activities of PPs are directly attributed to the phenolic hydroxyl groups that are good H-donating antioxidants, and then, they scavenge reactive oxygen species and break the cycle of generation of new radicals [2]. Thereby, the PPs inhibit the oxidation of lipids, proteins, and DNA. Furthermore, they inhibit the production of enzymes involved in radical-type generation, which are related to the development of diseases [7].

Phenolic compounds have been identified in a wide class of plant-derived foods such as fruits (berries and berry products), seeds, cereals, leaves, and vegetables, and the list is constantly growing [8]. The required mean daily PPs intake from fruits and vegetables among adults is 219 mg (males) and 193 mg (females) from fruit and 78 mg (males) and 67 mg (females) from vegetables [9]. However, there is a need for looking for new sources aiming to satisfy this intake and industry demand. Thereby, scientists have focused their attention on potential sources of PPs, including agro-food by-products, such as wastewaters (from olive, artichoke, maize, and winemaking processing industries), residues (orange press liquor, winery effluent, fruit seeds, fermented grape pomace, grape marc, etc.) and some other derivative by-products [10,11]. On the other hand, there is great interest in identifying tangible methods for extracting them from such sources, based on the limitation that the degradation of phenolic compounds generally occurs due to their low stability at high temperatures, presence of solvents, and long extraction times [12].

In this framework, the aim of this paper is to provide an overview of the conventional extraction and separation methods for phenolic-based compounds from different agro-food by-products, highlighting the role of tight ultrafiltration (UF) and nanofiltration (NF). The effect of key parameters on the performance of these processes in terms of productivity and selectivity towards target compounds are analyzed and discussed. Current extraction methodologies and pre-treatment systems before separation, fraction and concentration of target compounds are also analyzed in order to provide a wide outlook on the potential of tight UF and NF membranes in integrated systems for the production of functional ingredients.

## 2. Extraction Methodologies of Polyphenols (PPs)

Agro-residues produced during the handling and processing of fruits, vegetables and forest resources are characterized by a complex composition of phytochemicals such as vitamins, tocopherols, PPs, and carotenoids, along with complex carbohydrates and fiber which have been been associated with the alleged health-promoting effects of fruits and vegetables consumption. Therefore, their extraction from residues and agro-industrial by-products, as an alternative to the synthetic substances commonly used in the food, pharmaceutical, and cosmetic industries, has been strongly encouraged [13].

**Figure 2:**
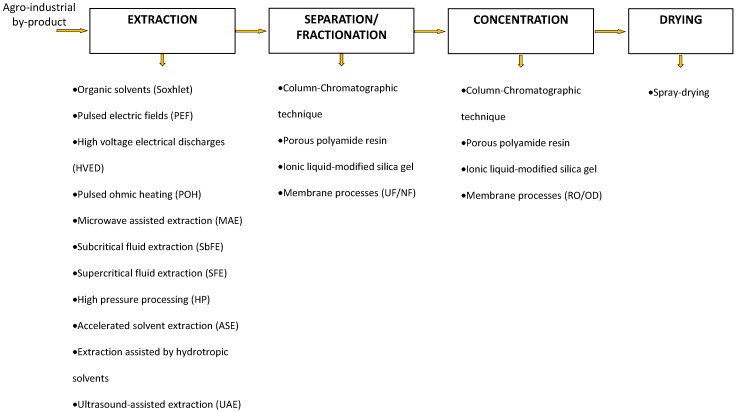
Layout of operation steps for recovering bioactive phenolic from agro-industrial by-products.

The development of adequate methods for the recovery of phenolic compounds from their original sources is crucial and can be accomplished through several steps (Figure 2). The processes employed should be environmentally sustainable and efficient, resulting in high yields.

Extraction is the first and crucial step and plays an essential role in the final characteristics of the product. The traditional process to extract PPs from by-products is the conventional extraction by solvent (Soxhlet extraction); however, drawback of leaving trace amounts of solvents or causing thermal degradation has driven to find new technologies to avoid that [14]. In this regard, several non-conventional processes have been reported as alternatives, much of them friendly in an environmental way, to improve the extraction of valuable compounds from agro-industrial by-products. Pulsed electric fields (PEF) [15], high voltage electrical discharges (HVED) [16], pulsed ohmic heating (POH) [17], microwave-assisted extraction (MAE) [18], subcritical fluid extraction (SbFE) [19], supercritical fluid extraction (SCFE) [20], high pressure processing (HP) [21], accelerated solvent extraction (ASE) [22], extraction assisted by hydrotropic solvents [23], and ultrasound-assisted extraction (UAE) [24] are typical examples.

Independent of the selected type of extraction, there are some common objectives, including selectivity and efficiency in the release of the target phytochemicals and a reproducible method regarding variations in the sample matrix [25]. Several factors are involved in the extraction yield. They include mixture components, such as the proportion of residue to be treated and the type of solvent used (water, acetone, ethyl acetate, alcohols as methanol, ethanol, and propanol, and their mixtures), as well as operating conditions such as temperature, pressure, extraction cycles, operating time, etc. The definition of the mixture and operating conditions will depend primarily on the residue characteristics, mainly by the type of PPs, and consequently by their solubility which will lead to the selection of specific solvents.

MAE is one of the most important and attractive non-conventional techniques to assist the extraction of phytochemicals from agro-industrial residues or by-products [18,26,27]. Several parameters have to be carefully selected to apply this technique for the extraction of phenolic compounds successfully. Solvent composition, solvent volume, extraction temperature and time, microwave power, and matrix characteristics (including particle size, sample-to-solvent ratio, and water content) are the most important [28]. Even though this technology has several advantages, its application at industrial scale is still limited due to factors related to capital costs as well as the recovery of nonpolar compounds and the loss of bioactivity of phytochemicals due to modification of their chemical structure [13].

UAE is considered a key technology in achieving the objective of a sustainable “green” extraction. It has been widely reported to have a significant effect on the rate of various processes in the chemical and food industry [29].

The ultrasound extraction mechanism involves two main types of physical phenomena: (i) diffusion across the cell wall, and (ii) the rinsing of cell contents after breaking the walls increasing mass transfer [30,31,32,33].

Several factors such as sonication time, temperature, and solvent selection as well as wave frequency and ultrasonic wave distribution should be controlled in order to maximize the extraction yield [34]. Based on the advantages of this technology (reduction of the processing time, energy consumption and uses of harmful and expensive solvents), several applications have been reported in the winery industry for extraction of PPs and anthocyanins from grapes [35], Cabernet franc grapes [17], grape by-products [36], red grape marc [33], grape seeds [37], and vine shoots [38].

Since its beginning in 1964, SCFE has attracted wide scientific interest because of its suitability for extraction and purification of compounds having low volatility or those which are susceptible to thermal degradation [14]. Supercritical fluids have liquid-like densities, higher diffusion coefficient, and low surface tension resulting in easy penetration of the supercritical solvent into the porous structure of the solid matrix to release the solute. The main advantages of supercritical fluids are: (i) have a higher diffusion coefficient and lower viscosity than liquids helping to a more favorable mass transfer, (ii) the absence of surface tension allowing their rapid penetration into the pores of solid matrices, which enhances the extraction efficiencies, (iii) an environment-friendly separation process without release of chemical residues.

Some applications for recovery of phytochemicals from food wastes have been reported in the literature, e.g., grape peel [39], tomato skin [40], orange peel [41].

Two to three decades ago, applications of pulsed electric fields (PEF) for treatment of liquid and solid foods became more and more popular [29]. The biological membrane of plant tissues is electrically pierced under the effect of PEF, therefore, they lose its semipermeability (temporally or permanently) in a process called electroporation. The effect of the aforementioned has opened great opportunities and application in the treatment of agro-industrial residues or by-products. In this sense, there exist many examples of its application for the processing of different raw materials such as crops, fruits, green biomass.

Even though there are differences between the technologies mentioned above regarding extraction yield, simplicity of operation, investment cost, operation time, safety, and degree of automation, all of them have comparative advantages in comparison to the traditional solvent extraction. Independent of the technology selected for the extraction, subsequent steps of separation, purification, and final concentration are needed. The next section will introduce conventional and non-conventional separation methodologies.

## 3. Separation and Purification Methodologies of PPs

The obtained extract, whatever the extraction methods used, have not only bioactive phenolics and PPSs but also others components such as mono- and poly-saccharides, as well as the solvent used for the extraction. Therefore, a separation step is crucial not only for removing the solvent but also to purify bioactive compounds.

Traditional methods of polyphenol purification comprises coagulation and precipitation of impurities (use of chemicals, solvents), adsorption of PPs on resins, elution of purified PPs, and their concentration by solvent evaporation.

Adsorption, ion exchange, column chromatography, and affinity chromatography can be grouped as recovery techniques in which the removed compound or solute establishes an equilibrium between sites on a solid phase material and the solution. In adsorption, the removed species is bonded to the solid phase material by polarity or weak chemical bonds. Ion exchange recovers material by the interchange of ions between the liquid and solid phases. Column chromatography may use adsorptive, ion exchange or molecular sieve materials to separate solutes which are first loaded onto a column of the separation material and then eluted in such a manner that the individual solutes are collected in separate fractions. In affinity chromatography, the removed species is bound with a high level of selectivity to ligands covalently attached to a solid matrix [42].

These operations account for a significant part of the total costs of polyphenol production. Additionally, some of them are based on a phase change which implies the application of temperature that can produce a decrease in the healthy activities of phytochemicals.

In this regard, pressure-driven membrane processes such as microfiltration (MF), ultrafiltration (UF), nanofiltration (NF) and reverse osmosis (RO) are consolidated systems in various productive sectors, since the separation process is athermal and does not involve phase changes or chemical agents [43]; additionally, they are characterized by high efficiency, simple equipment and low energy consumption [44]. In a typical PPs recovery process, these processes can be coupled with different extraction techniques and/or combined between them in integrated systems in order to remove the organic solvent, large (proteins, polypeptides) and small (salts, simple sugars) molecules as well as other impurities with the aim of producing relatively pure mixtures of active compounds.

## 4. General Aspects of Microfiltration (MF), Ultrafiltration (UF) and Nanofiltration (NF) Processes

Membrane-based technologies, such as MF, UF and NF, are physical separation processes allowing the separation of different compounds from a feed solution under a hydrostatic pressure difference applied between the two sides of a permselective barrier. As a result, the feed solution is divided into a permeate fraction containing all components that have permeated the membrane and a retentate fraction containing all compounds rejected by the membrane, within some of the solvent [45,46]. The separation is determined by the pore size-range of the membrane, which is mainly related to its molecular weight cut-off (MWCO), and to a lesser extent on molecular shape, charge and hydrophobicity. Typically, a membrane’s MWCO refers to the smallest average molecular mass of a known solute that will not effectively diffuse across the membrane [43].

MF membranes have large pore sizes ranging from 100 to 10,000 nm (separating particles), while UF membranes have pore sizes between 2 and 100 nm which are able to retain molecules with molecular weights ranging from 350,000 to 1000 Da. NF membranes are characterized by narrow pore sizes from 0.5 to 2 nm, retaining micromolecules with molecular weights from 120 to 1000 Da [45].

**Figure 3:**
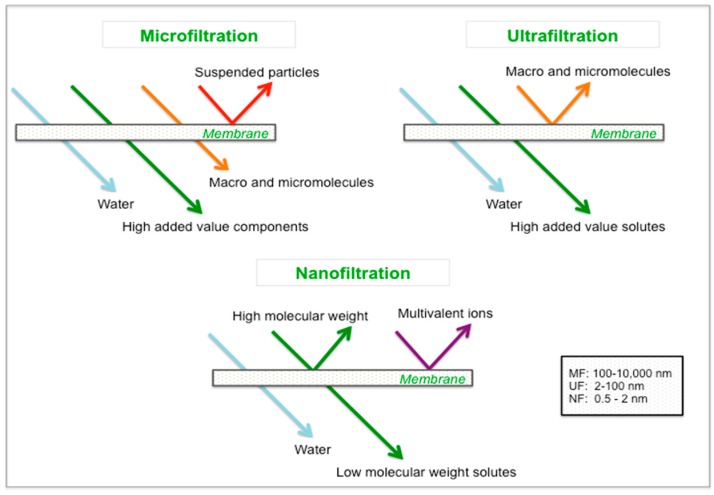
Schematic description of MF, UF and NF membranes (adapted from [12]). MF, ultrafiltration; UF, ultrafiltration; NF, nanofiltration.

A schematic representation of MF, UF and NF processes is illustrated in Figure 3. Typical operating pressures related to these processes are specified in Table 1.

Wide UF membranes, with MWCO ranging between 50 and 100 kDa, can fundamentally be used to recover different types of macromolecules (such as suspended solids, carbohydrates, proteins, and pectins). UF membranes from 4 to 30 kDa are effective to concentrate high molecular weight components (such as tannins, proteins, hydrolysates, and some high molecular weight phenolic fractions), while tight UF membranes, from 1 to 3 kDa, are highly effective to concentrate low molecular weight compounds (such as anthocyanins, low molecular weight PPs, low molecular weight sugars, and peptides); indeed, these membranes are in the molecular limit of the NF process [47]. Low nominal MWCO NF membranes (ranging from 350 to 400 Da) can practically retain the same molecules recovered by tight UF membranes (1–3 kDa); however, high separation efficiency in NF membranes is expected. On the other hand, fine NF membranes (120–300) are the best ones to selectively recover and concentrate low-molecular weight compounds (like low molecular weight PPs). Thus, tight UF and NF membranes are the most suitable ones to meet the recovery of PPs.

**Figure 4:**
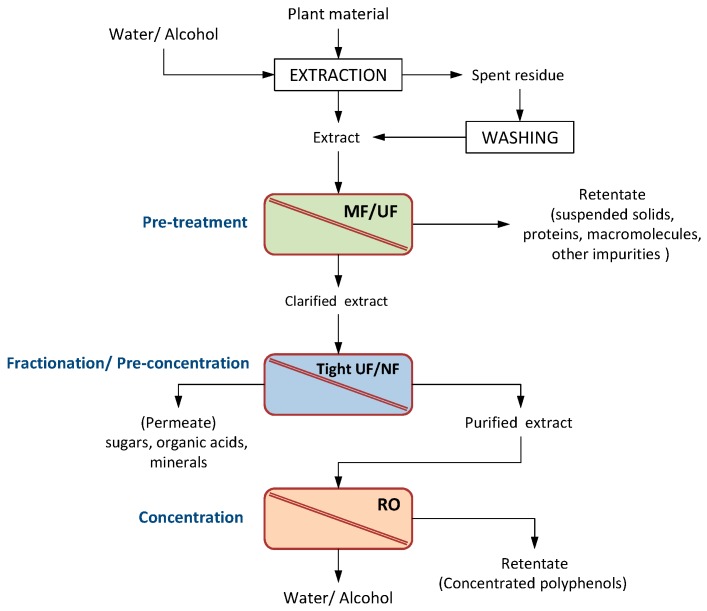
General flow sheet for the recovery of phenolic compounds from vegetable sources by membrane processing (MF, ultrafiltration; UF, ultrafiltration; NF, nanofiltration; RO, reverse osmosis).

The general flow-sheet for the recovery, purification and concentration of phenolic compounds from vegetable sources through a combination of membrane operations is illustrated in Figure 4. Water or organic solvents are used for the extraction of the target components from the plant material. A pre-treatment step based on the use of UF or MF membranes is used to retain suspended solids and large molecules such as proteins and polypeptides and other impurities. The UF permeate containing PPs is fed to tight UF or NF membranes to separate phenolic compounds from low molecular weight compounds. RO can be used to further concentrate the permeate and to facilitate the removal of the solvents.

## 5. Recovery of PPs by Tight UF and NF Membranes

In principle, separation processes using tight UF and NF membranes are able to separate specific compounds through a sieving mechanism based on the MWCO; however, the membrane’s MWCO is not the only criterion that has to be taken into account. For instance, the asymmetric manufacture of membrane pores does not always reflect a narrow MWCO range; in addition, some other phenomena (e.g., concentration polarization, membrane fouling, coulombic and hydrophobic interactions) also contribute to the phenolic retention [12,47,48]. In the following sections, the influence of membrane material, MWCO and operating parameters on the recovery of PPs by tight UF and NF membranes is analyzed and discussed together with the contribution of specific pre-treatments of feed solutions aimed at limiting the formation of fouling layers which are the main cause of reduced membrane performance.

### 5.1. Effect of Pre-Treatment on the Membrane Performance

Agro-food wastewaters contain considerable amounts of high molecular weight solutes in the shape of suspended solids and colloidal particles identified as detrimental from a bioactivity perspective. Physical and physico-chemical processes (screening, sedimentation, centrifugation, adsorption, etc.) are used as primary separation (as pre-treatment) to remove suspended solids or other impurities. Because of this purpose, the use of UF and MF processes has been often introduced in integrated systems for recovery of phytochemicals from agro-industrial by-products as preliminary step, in alternative to the use of conventional systems based on the use of adsorbents and filter aids, before a fractionation step performed by using tight UF and/or NF membranes [49]. The general flow-sheet for the recovery of phenolic compounds from vegetable sources is illustrated in Figure 4. Typical selected applications are reported in the following.

#### 5.1.1. Olive Mill Wastewaters (OMWs)

The olive oil extraction is a water-intensive process that generates a huge quantity of polluted effluents commonly referred to as olive mill wastewaters (OMWs). These effluents are characterized by high concentrations of organic compounds, including organic acids, sugars, tannins, pectins and phenolic substances that make them phytotoxic and inhibit bacterial activity. The typical composition of OMWs is reported in Table 2.

Suspended solids of raw OMWs can be removed by centrifugation. The following treatment by UF allows a complete separation of fats, completely rejected by the membrane, from salts, sugars and polyphenols, contained in the permeate. A chemical oxygen demand (COD) reduction of about 90% is reached through the combination of both processes [51]. Similarly, the prefiltration of raw OMWs with a polypropylene screen (80 μm) followed by treatment with multichannel ceramic UF membranes (pore size 100 nm), produces a separation of high molecular weight constituents including fats, lipids and suspended solid particles [52].

Cassano et al. [50] evaluated the performance of an integrated membrane process based on the use of UF and NF membranes in the recovery of phenolic compounds from OMWs. The UF pre-treatment produced a lower permeate flux decay and higher steady-state permeate flux values in the next UF step performed with a composite fluoro polymer membrane of 1 kDa (Etna 01PP, from Alfa Laval, Nakskov, Denmark).

In a previous study [53], the pre-treatment of raw OMWs with a ceramic MF membrane, before a NF step, permitted to achieve a reduction of total suspended solids (TSS) and total organic carbon (TOC) of 91% and 26%, respectively. In the MF permeate, were recovered 78% of PPs; therefore they were purified from suspended solids and, partially, from other organic compounds. The rejection of the MF membrane towards different analyzed low molecular weight PPs was between 7.2% (protocatechuic acid) and 27.7% (oleuropein).

An innovative process design for PPs encapsulation from OMWs was proposed by Bazzarelli et al. [54] through a combination of conventional pressure-driven processes (MF and NF) and relatively new membrane operations such as osmotic distillation (OD) and membrane emulsification (ME). After the removal of suspended solids by an acidification/MF step, the MF permeate was processed by NF in order to obtain water from the permeate side and a concentrated polyphenolic solution from the retentate side. The MF process had a negligible rejection towards phenolic compounds (about 6.8%) assuring their recovery in the permeate stream.

According to Arvaniti et al. [55], UF provides a “clean” solution appropriate to feed next treatment processes based on the use of NF or RO membranes in the recovery of phenolic compounds from OMWs. UF alone cannot isolate individual phenolic fractions but without UF further purification with the NF and/or RO membranes cannot be achieved.

Standardized fractions enriched in phenolic compounds were obtained from *Olea europaea* L. tissues (leaves and pitted olive pulp) and *Cynara scolymus* L. by-products (leaves and stems) through an environmentally friendly process based on a water extraction and membrane separation technology [56]. In the investigated approach a preliminary MF step was carried out with tubular ceramic membranes in titanium oxide to remove suspended solids improving the performance of following steps performed with spiral-wound membrane modules in polyethersulfone (PES).

#### 5.1.2. Artichoke Wastewaters

The artichoke (*Cynara scolymus* L.) processing industry generates large amounts of agricultural solid wastes (leaves, stems, bracts of the artichoke plant) and wastewaters. These wastes are considered a rich source of bioactive phenolic compounds (with mono- and di-caffeoylquinic acids being the major components) and also inulin, fibres and minerals [57]. The typical composition of artichoke wastewaters is reported in Table 3.

The use of PES hollow fiber UF membranes with a MWCO of 50 kDa allowed to produce a clear solution depleted in suspended solids and enriched in phenolic compounds from raw artichoke extracts [58]. Similarly, ceramic UF membranes of 15 kDa produced a clarified fraction depleted in suspended solids and macromolecular compounds from artichoke wastewaters produced in the blenching step [59]. Cassano et al. [60] reported the use of cellulose triacetate UF membranes with MWCO of 150 kDa in hollow fiber configuration (FUC 1582, Microdyn-Nadir, Wiesbaden, Germany) for the removal of suspended solids from artichoke brines. In all these approaches the use of UF allowed to reduce fouling phenomena in the subsequent NF process.

**Figure 5:**
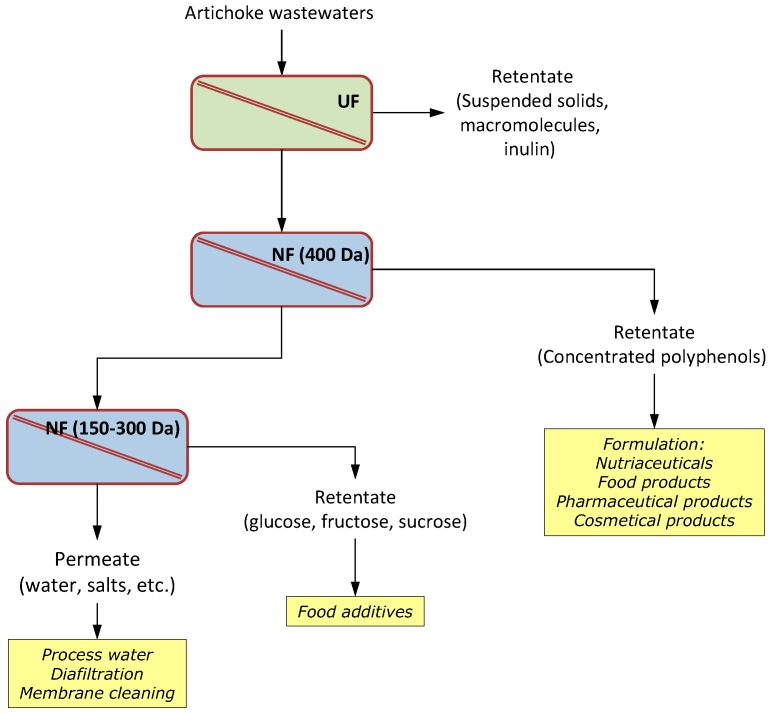
Proposed flow sheet for the recovery of phenolic compounds and sugars for artichoke extracts (adapted from [58]).

The proposed flow-sheet for the recovery of phenolic compounds from artichoke extracts is illustrated in Figure 5.

#### 5.1.3. Citrus by-Products

The citrus juice processing industry generates large amounts of by-products such as peels and seed residues which may account for up to 50% of the total fruit weight. Most of the waste residue from commercial juice extractors is shredded, limed, cured and pressed into press liquors and press cakes which are then processed independently. Press liquors are a complex mixture containing soluble sugars (sucrose, glucose and fructose), insoluble carbohydrates, fibers, organic acids, essential oils, flavonoids and carotenoids [61]. The typical composition of orange press liquor is reported in Table 4.

The UF process separates the flow from the press liquor into a permeate with a total soluble solids content and an acidity level similar to that of the feed solution and a retentate containing suspended solids such as proteins and fibers and high molecular weight carbohydrates such as pectins associated with cloud [63]. MF polyvinylidene fluoride (PVDF) membranes in hollow fiber configuration and with pore size of 0.22 μm allowed to remove all suspended solids from orange press liquor preserving phenolic compounds in the clarified liquor [62]. Cassano et al. [64] investigated the recovery and concentration of flavonoids from orange press liquor by using a combination of membrane operations such as UF, NF and OD. Suspended solids were removed from the raw press liquor by using polysulfone (PS) hollow fiber membranes with MWCO of 100 kDa; flavonoids and anthocyanins were recovered in the clarified liquor according to the low rejection of the membrane towards these compounds (lower than 1%).

Bergamot (*Citrus Bergamia* Risso) is a natural citrus fruit widely exploited for the production of essential oil employed in pharmaceutical, cosmetic or food industry. The juice has not found a real use in the food industry due to its bitter taste and it is considered just a secondary or even waste of the essential oil extraction; however, it has been recognized as a source of bioactive compounds, including flavonoids with statin-like properties (named brutieridin and melitidin) able to reduce cholesterol levels in blood [65].

Integrated membrane processes have been investigated for the recovery of phenolic compounds from bergamot juice [66,67]. In these approaches the raw juice was depectinised in order to hydrolyse both high and low esterified pectins and ultrafiltered with hollow fiber membranes (PS, 100 kDa, from China Blue Star Membrane Technologies, Beijing, China) in order to produce a clear juice depleted in suspended solids. The clarified juice was then submitted to a fractionation/concentration step by using tight UF and/or NF membranes.

### 5.2. Effect of Operating Conditions on the Membrane Performance

The membrane performance is influenced by a series of factors which should be controlled not only to maximize the permeate flux but also to improve selectivity towards target compounds [68]. Transmembrane pressure (TMP) plays an important role in the membrane performance because it has a direct effect on membrane fouling which can be reversible or irreversible [69]. Increasing TMP produces a linear increase in the permeate flux; however, there is a critical point, defined as critical transmembrane pressure at which this linear relationship is lost. The limiting point is where the maximum flux value is obtained under some operating conditions and it cannot be increased by varying TMP. Therefore, to keep a continuous process the TMP selected must be lower than the critical value, this means work under the critical zone in which the fouling effect is minimum.

The rejection of phenolic compounds usually increases by increasing the TMP [70,71]. This phenomenon can be described by the so-called film layer theory assuming the formation of a thin layer of a specific thickness in the zone adjacent to the membrane surface where the concentration decreases from the surface to the bulk. At higher TMP values concentration polarization and fouling phenomena are more severe, leading to the formation of an additional selective layer on the membrane surface increasing the retention coefficient.

**Figure 6:**
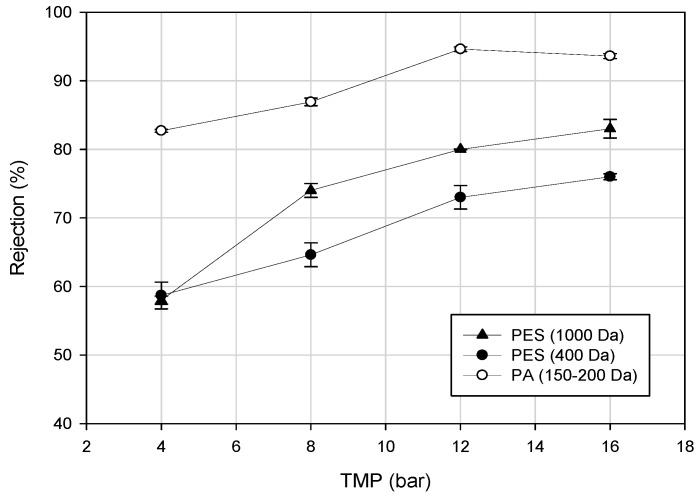
Rejection of NF membranes towards total phenols of clarified bergamot juice as a function of applied pressure [67].

The rejection of total PPs for different NF membranes in the treatment of clarified bergamot juice is shown in Figure 6. The increase of operating pressure in the range between 4 and 16 bar for the selected membranes resulted in an increased rejection of phenolic compounds [67]. Giacobbo et al. [72] found a linear relationship between TMP and rejection of phenolic compounds (in the range 3–15 bar) in the treatment of second racking wine lees by using a composite fluoro polymer UF membrane with a MWCO of 1 kDa (Etna 01PP, from Alfa Laval).

The increasing of the operating pressure enhanced the effects of fouling in the treatment of ultrafiltered OMWs by NF membranes [73]. For all investigated membranes (NF270, NF 90 and NF-self-made) rejection coefficients towards phenolic compounds increased by increasing the operating pressure in the range of investigated values (5–20 bar).

A different trend was observed in the treatment of an aqueous extract from distilled grape pomace by using a spiral-wound UF membrane with a MWCO of 1 kDa (GE2540 from GE Osmonics, Minnetonka, MN, USA): in this case the retention of phenolic compounds decreased by increasing the TMP from 2 to 8 bar [70].

In general, higher temperatures will lead to higher flux in both the pressure-controlled region (under the critical TMP) and in the mass transfer-controlled region (upper the critical TMP). The effect produced by increasing temperature is related to a decrease in the viscosity of the feed, which imply lower pumping energy and horsepower required [74]. In any case, when membrane processes are applied to the recovery of phenolic compounds, the temperature of operation should be as lower as possible to avoid loss of compounds bioactivity.

The retention of a cross-linked aromatic polyamide (PA) membrane with a MWCO of 150–300 Da (Desal DK, from GE Osmonics) towards phenolic compounds of artichoke brine at different temperatures showed no significant difference among the investigated compounds. Therefore, a higher flux can be obtained by increasing the operating temperature without affecting the final composition of the concentrated product [60].

An increase in feed flow rate enhances mass transfer coefficient, reduces concentration polarization and accumulation of solutes on the membrane surface [75]. In this sense, agitation and mixing of the fluid near the membrane sweep away the accumulated solute deposited on the membrane surface, reducing the hydraulic resistance of the “cake” and the thickness of the boundary layer.

Giacobbo et al. [76] evaluated the recovery of polysaccharides and polyphenols from winery effluents by using a PES flat-sheet UF membrane with a MWCO of 2000 Da (GR95PP, from Alfa Laval). For all the investigated feed circulation velocities a linear variation of the permeate flux with TMP was observed. The slope of this variation was identical for low feed circulation velocities (0.6 and 0.44 ms^−1^) and well below the one corresponding to the highest velocity (0.87 ms^−1^).

The use of air sparging via the injection of gas bubbles into the feed stream, applied successfully to the enhancement of UF and MF processes, has also been investigated in the treatment of molasses wastewater by NF [77]. Both concentration polarization resistance and osmotic pressure resistance decreased with cross-flow velocity, but increased with feed concentration and the operating pressure. An appreciable reduction of concentration polarization resistance in presence of gas sparging was observed.

The concentration of phenolic compounds in the retentate fraction increases by increasing the volume reduction factor (VRF) of the filtration process. Cassano et al. [60] observed an increasing concentration of chlorogenic acid and caffeoylquinic acid derivatives by increasing the VRF in the NF of artichoke brine by using a cross-linked aromatic polyamide (PA) membrane with a MWCO of 150–300 Da (Desal DK, from GE Osmonics). However, the concentration of phenolic compounds results were not proportional to the VRF used. Different factors, including the surface morphology, the pore size distribution and adhesion in membrane may contribute to this phenomenon [78].

### 5.3. Effect of Molecular Weight Cut-off (MWCO) on the Membrane Performance

The separation mechanism involved in UF and NF processes seems to be simple in theory because it is based on a “sieving effect” which allows compounds to be retained on the basis of their molecular weight (MW). However, in practice, the MWCO of the membrane is not an absolute barrier and the solute rejection/transport through the membranes is also influenced by interactions between the membrane itself and the compound being filtered. Another aspect is that the manufacture of the membrane creates asymmetric pores that do not always reflect the respective range of the MWCO [47]. A reduction of the MWCO of the membrane is also observed during polarization concentration and fouling phenomena. In addition, PPs tend to interact and bind non-covalently to proteins and polysaccharides [79]. This implies that low MW PPs can be concentrated in the retentate, following the structural characteristics of high MW PPs.

The effect of MWCO of tight UF and NF membranes on the purification of PPs from other biologically active compounds has been studied by different authors. For instance, Conidi and Cassano [67] evaluated two different spiral wound PES membranes with MWCO in the range of 300–1000 Da to separate PPs from sugars in ultrafiltered bergamot juice. The NF PES 10 membrane with a MWCO of 1000 Da (from Microdyn-Nadir) produced the best separation of PPs from sugars when compared with the N30F membrane (PES, 300 Da, from Microdyn-Nadir). Results obtained with the N30F membrane showed that the rejection of the membrane towards flavonoids was of 85–90%, while the rejection towards total antioxidant activity (TAA) and PPs was of about 70–76%. The observed rejection towards sugar compounds was 65%. This type of membrane allowed only a partial purification of flavonoids from sugars; on the other hand, the measured rejection of the NF PES10 membrane towards flavonoids was between 88.4% and 90.1%, while the observed rejection towards sugars was of 35%. These results could be explained by steric effects, physicochemical interactions with the membrane or adsorption of phenolic compounds on membrane surface. These values also confirmed the high fouling index measured for this membrane.

**Figure 7:**
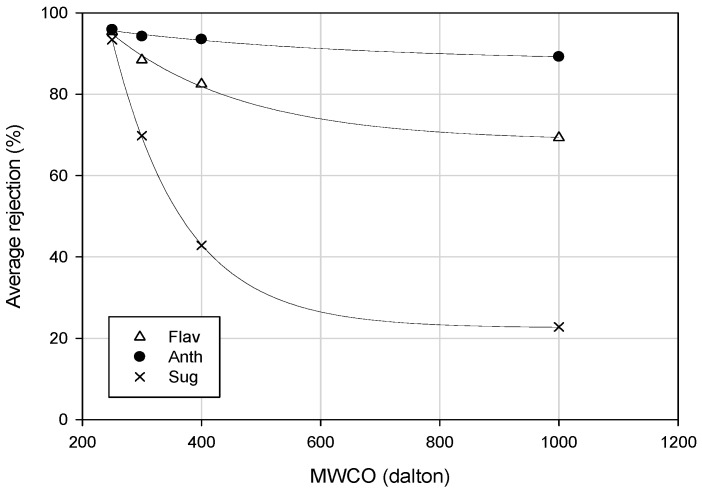
Nanofiltration of orange press liquor. Average rejection values of flavonoids, anthocyanins and sugars as a function of MWCO [80].

Similarly, four spiral wound membranes of different materials (PA, polypiperazine amide and PES) and MWCOs (180, 300, 400 and 1000 Da) were employed in the separation of phenolic compounds of orange press liquor from sugar compounds [80]. A correlation between sugar rejection and MWCO was identified. Indeed, a strong reduction in the average rejection of sugars was observed when MWCO was increased. On the other hand, the rejection of anthocyanins was higher than 89%, independent of the MWCO of the selected membrane. The PES10 NF membrane showed the lowest average rejection of sugar compounds (22%) and high rejections of anthocyanins (89.2%) and flavonoids (70%), indicating that this membrane showed the best separation of PPs from sugars (Figure 7).

**Figure 8:**
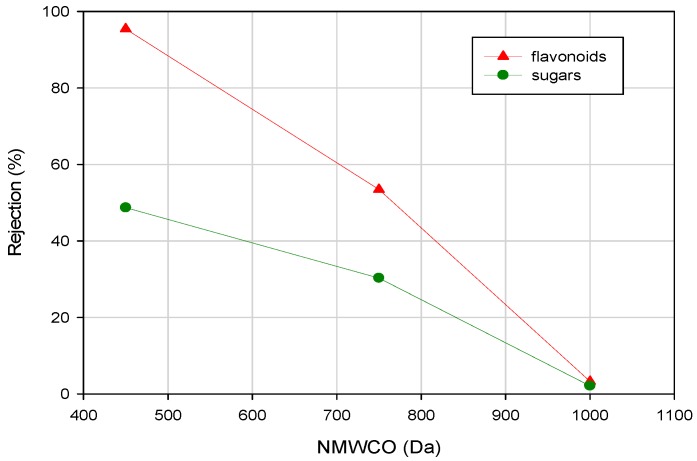
Effect of nominal MWCO on the rejection of sugars and flavonoids by UF and NF membranes [66].

In a previous study, the same authors tested different UF and NF membranes and their performance in the separation and concentration of PPs from ultrafiltered bergamot juice [66]. In particular, the ultrafiltered juice was treated with a UF membrane (Etna 01PP, from Alfa Laval) and two different ceramic NF membranes (monotubular TiO_2_ membranes, 750 and 450 Da from Inopor) in order to evaluate the effect of the MWCO on the rejection by the membranes towards sugars, organic acids and PPs. The effect of MWCO on the rejection of sugars and flavonoids is illustrated in Figure 8.

Experimental results indicated that the Etna 01 PP membrane preserved flavonoids and sugars in the permeate fraction in agreement with the MWCO of this membrane (1000 Da) and the MW of the investigated compounds which is in the range 180–610 Da. The NF 750 Da membrane showed a rejection towards flavonoids in the range of 43–62% and a rejection towards PPs of 44%. Therefore, phenolic fractions with molecular weight greater than 750 Da were retained on the retentate side of the membrane. Besides, flavonoids identified in the juice (naringin, narirutin, hesperidin and neohesperidin), with molecular weights from 550 to 610 Da, were also partially retained by the membrane due to fouling phenomena. The observed rejection towards sugars was about 30%. The rejection of the NF 450 Da membrane towards flavonoids was in the range of 91–99%. Therefore, flavonoids were retained on the retentate side of the membrane, in agreement with the MW of the analyzed flavonoids. The rejection of this membrane towards sugars was 48% indicating a better performance in terms of separation between sugars and flavonoids in the clarified juice.

Giacobbo et al. [72] investigated the use of UF and NF membranes aiming the fractionation of polyphenols and polysaccharides present in wine lees. In particular, composite fluoro polymer UF membranes with MWCO of 1 and 10 kDa (Etna 01PP and Etna 10PP, from Alfa Laval) and a polypiperazine NF membrane of 200–300 Da (NF 270 from Dow-Filmtec, Midland, MI, USA) were used. In agreement with the MWCO of the selected membranes, the NF 270 showed a rejection higher than 90% towards total PPs and anthocyanins, while the rejection of the 10-kDa membrane was lower than that measured for the 1-kDa membrane (rejections were of about 50–60%, respectively). The rejection of the NF 270 membrane towards polysaccharides was of 99%, while for both UF membranes was higher than 77%. Experimental results indicated the possibility to fractionate PPs and polysaccharide mixtures by UF through the production of a retentate fraction enriched in polysaccharides and a permeate stream enriched in anthocyanins and other PPs.

For PES membranes (NP030 and NP010) the rejection towards phenolic compounds of artichoke brines (caffeoylquinic acids, flavonoids, chlorogenic acid and cinarin) is a function of the MWCO; an increased rejection for all these compounds was observed for the NP030 membrane which is characterized by a lower MWCO [60].

Tylkowski et al. [81] studied the influence of the MWCO on the rejection of total phenols and flavonoids of an ethanolic extract from *Sideritis* ssp. L. At this purpose, three flat-sheet organic solvent resistant NF membranes (StarmemTM 240, DuramemTM 500 and DuramemTM 300, all from Membrane Extraction Technology, London, UK), with MWCO in the range of 200–500 Da, were tested. Experimental results indicated that all membranes showed high rejection towards flavonoids (from 97% up to 99%) as confirmed by the low concentration observed in the permeate. The best purification of flavonoids from low molecular weight PPs was possible with the membrane having MWCO higher than 400 Da, while the 300 Da membrane allowed a complete concentration of these compounds (up 3–4 times with respect to the starting solution).

Pinto et al. [82] evaluated the performance of different UF and NF membranes, in terms of phenolic compounds and carbohydrate rejection, in the treatment of ethanolic extract of *Eucalyptus globulus* bark. Among the investigated membranes JW (30 kDa, PVDF from GE Osmonics), PLEAIDE (5 kDa, PES, from Orelis, Salindres, France) and 90801 (350 Da, PA derivative from SolSep, Apeldoorn, The Netherlands) were selected for the concentration process of bark extract. The rejection values for total phenolic compounds increased in the order JW, PLEAIDE, 90801, following the trend of the MWCO. Lower rejections were found for total carbohydrates indicating the suitability of the UF and NF processes for the removal of sugar moieties in the permeate stream.

Recently, different tight UF and NF membranes in flat-sheet configurations, with MWCO ranging from 1000 to 4000 Da were tested to purify PPs from sugars in clarified pomegranate juice [83]. According to the experimental results, the Desal GK membrane, in thin film composite, with a MWCO of 2000 Da (from GE Osmonics) showed high retentions towards anthocyanins, total PPs and total antioxidant activity (TAA) (in the range 80–95); on the other hand, rejection values of 1–3% towards fructose and glucose, were measured (Table 5). An improved purification of PPs was obtained combining the concentration step with diafiltration in a discontinuous way.

### 5.4. Effect of Membrane Material on the Membrane Performance

The selection of the membrane materials is extremely important for assuring good membrane stability in the presence of the main solvent and a low adsorption of solutes. In order to avoid solute adsorption and fouling, the membrane should exhibit low affinity towards the solutes. On the other hand, a high affinity towards the membrane, ensures high fluxes of solvent.

An interaction between the membrane structure and the MWCO with the polarity of smaller solutes has been also reported. For instance, during UF of winery sludge extracts with membranes of MWCO of 100 kDa and 20 kDa (GR40PP and GR70PP, both from Alfa-Laval), phenolic compounds and sugars were concentrated in the retentate side (rejection in the range 50–99%) despite their low corresponding molecular weight (<1000 Da) [84]. The high retentions of low MW components by large membrane pores has been attributed to polarity characteristics. Similarly, high retentions of PPs were measured for UF membranes employed in the concentration of grape seed extracts. In particular, a PES membrane of 150 kDa and a PVDF membrane of 50 kDa (UP150 and UV050, respectively, from Microdyn-Nadir) showed a rejection towards PPs of 87% and 91%, respectively [85].

Benitez et al. [86] reported the use of two different PES UF membranes with MWCO of 20 and 50 kDa (PW and PT, respectively, from GE Osmonics) for the recovery of ellagic acid from cork wastewaters. The selected membranes showed a rejection higher than 90% towards this component; however, the PT membrane with lower MWCO showed a higher rejection when compared with the PW membrane.

In a different approach, PVDF UF flat sheet membranes with different MWCO (in the range of 10–1000 kDa) were investigated for the fractionation of PPs on the basis of their MW from Concord grape (*Vitis labrusca* L.) juice [87]. The selected membranes were also compared in terms of productivity, fouling index and antioxidant potential. Experimental results indicated that UF membranes with MWCO lower than 100 kDa permitted a concentration of polymeric anthocyanins in the retentate fractions, while monomeric anthocyanins decreased in the retentate fractions of these membranes. This behavior was attributed to the possible presence of anthocyanins deposition and pore size restriction or gel layer formed, which would increase the resistance to the mass transfer or the association of different classes of anthocyanins (e.g., hydrogen bonding).

According to the results obtained by Russo [88] in the fractionation of OMWs with pressure-driven membrane operations, UF purifies phenolic compounds contained in the MF permeate but ceramic 1 kDa and polymeric 6 kDa membranes did not show different selectivity among the polyphenolic molecules but only different values of rejection and yield. In particular, the UF 6 kDa showed a rejection of about 45% for free PPs, 75% for oleuropein and 45% for hydroxytyrosol. The rejection of 1 kDa membrane towards free PPs and hydroxytyrosol was of about 31–32%.

Regenerated cellulose UF membranes with MWCO of 5 and 10 kDa exhibited lower retention of phenolic compounds in the treatment of OMWs when compared with PES membranes of 4 and 10 kDa [89]. This behavior was attributed to polar interactions, such as van der Waals and electron donor-acceptor interactions, and multiple hydrogen bonds between polyvinylpyrrolidone (PVP), commonly used as additive in the manufacture of PES membranes, and PPs. The higher fouling index of PES membranes in comparison to PA membranes was also attributed to the higher adsorption of phenolic compounds on PES membranes [90]. Similarly, Boussu et al. [91] observed a higher adsorption of uncharged organic compounds on PES membranes, in comparison with PA membranes. The volume fraction of small and large pores, rather than the contact angle, was considered as the most influential effect on the adsorption of organic compounds. Therefore, a low volume fraction of small pores in the top layer should minimize membrane fouling remarkably.

Steric exclusion and hydrophobic attraction have been proposed as the main interactions occurring between phenolic compounds and semiaromatic piperazine-based PA membranes [92]. The hydrophobicity of compounds with molecular weight below the MWCO of a thin-film composite PA membrane of 200 Da (NF90, Dow-Filmtec) was considered the most important parameter affecting the adsorption of phenolic compounds on the membrane surface [93]. For this membrane authors investigated also the influence of type and position of the substituted functional groups in the benzene ring [94]. A systematic decrease in relative flux was obtained when the additional functional group on the aromatic ring shifted from hydroxyl to chloro and finally to nitro. The membrane retention increased following the sequence para → meta → ortho, while for polyhydric phenols, the retention increases according to the sequence: mono → di → tri. In both cases, steric hindrance was recognized as the main factor determining the membrane performance.

This membrane exhibited high retentions (about 97%) towards phenolic compounds of aqueous extracts of pequi (*Caryocar brasiliense* Camb.) fruit. On the other hand, the retention for alcoholic extracts were much lower (of the order of 15%). This behavior was attributed to the predominant hydration by water molecules in water systems which reduces the effective membrane pore size due to hydration/solvation of pore walls [95].

The high rejection of NF polymeric membranes towards anthocyanins of citrus press liquor (see Figure 6) was attributed to the positive charge of anthocyanins at the pH of the liquor which was of 3.4. At this pH, all the selected membranes exhibit a positive charge. Consequently, the electrostatic repulsion, independent of the MWCO of the selected membranes, contributed to the high observed rejection (more than 89%) [80].

## 6. Conclusions

Fruit and vegetable processing residues are considered highly polluting wastes because of their high organic load and phytotoxic and antibacterial phenolic substances resistant to biological degradation. On the other hand, intensive research in the field of agro-food waste management suggests that these effluents should be regarded as a useful resource for the recovery of fine chemicals and for different biotechnological applications such as the production of important metabolites. In particular, agro-industrial by-products can be considered a promising alternative to synthetic antioxidants used in food, cosmetics and pharmacy since they are an inexpensive source of valuable compounds, mainly polyphenols which are known for their antioxidant properties.

Pressure-driven membrane operations are well-known established technologies for the treatment of high strength wastewaters aimed at the production of purified water for recycle or reuse and the recovery of valuable compounds.

The use of tight ultrafiltration and nanofiltration membranes has been reviewed in the light of their growing use for the separation and purification of phenolic compounds from agro-food by-products and extracts. Tailor made processes for specific by-products can be identified through an appropriated selection of membrane typology as well as optimization of operating and fluid-dynamic conditions which are key parameters influencing both productivity and selectivity towards target compounds.

The combination of tight UF and NF membranes with conventional extraction systems and/or membrane pre-treatment of raw agro-food wastewaters offer new opportunities for the formulation of nutraceutical products while reducing the environmental pollution of these effluents.

## Figures and Tables

**Table 1:** Pressure requirements and separation mechanisms of MF, UF and NF (adapted from [45]).

Membrane Process	Required Pressure (Bar)		Typical Separation Mechanism
	Min.	Max.	
**Microfiltration**	0.1	2	Sieving
**Ultrafiltration**	0.1	7	Sieving
**Nanofiltration**	3	25	Sieving & charge effect

**Table 2:** Typical composition of olive mill wastewaters [50].

pH	5.0
Total soluble solids (g/kg)	29.0
Total suspended solids (%, *w*/*w*)	3.8
Total organic carbon (mg/L)	13,436
Total inorganic carbon (mg/L)	10.0
Total phenols (mg/L gallic acid)	1409
Hydroxyl-tyrosol (mg/L)	3.8
Protocatechuic acid (mg/L)	25.0
Catechol (mg/L)	7.5
Tyrosol (mg/L)	39.0
Caffeic acid (mg/L)	5.0
*p*-cumaric acid (mg/L)	1.0

**Table 3:** Typical composition of artichoke wastewaters [58].

Total Suspended Solids (%, *w*/*w*)	2.5 ± 0.1
Total soluble solids (g/kg)	30.5 ± 0.5
Glucose (mg/L)	960.0 ± 1.0
Fructose (mg/L)	837.0 ± 1.1
Sucrose (mg/L)	1050.0 ± 0.4
TAA (mM Trolox)	8.00 ± 0.04
Chlorogenic acid (mg/L)	251.0 ± 2.6
Cynarin (mg/L)	164.7 ± 1.41
Apigenin-7-*O*-glucoside (mg/L)	101.0 ± 2.0

**Table 4:** Typical composition of orange press liquor [62].

Suspended Solids (%)	8.3 ± 0.2
TSS (°Brix)	18.0 ± 0.1
pH	3.5 ± 0.2
TAA (mM Trolox)	21.4 ± 3.5
Total polyphenols (as GAE) (ppm)	1217.3 ± 57.0
Neohesperidin (ppm)	20.7 ± 0.4
Hesperidin (ppm)	18.4 ± 0.3
Naringin (ppm)	5.5 ± 0.1

**Table 5:** Rejection coefficient (expressed as %) of UF and NF membranes towards sugars and phenolic compounds of pomegranate juice (adapted from [70]).

Analysed Compounds	Membrane Type			
	Etna 01 PP (1000 Da)	PES004 (4000 Da)	MPF-36 (1000 Da)	Desal GK (2000 Da)
Glucose	3.31 ± 4.96	4.18 ± 0.46	3.86 ± 1.20	1.61 ± 0.01
Fructose	2.36 ± 0.04	3.17 ± 0.84	9.92 ± 1.11	2.17 ± 0.84
Total polyphenols	85.22 ± 0.23	94.97 ± 0.01	97.50 ± 0.02	88.25 ± 0.34
TAA	57.11 ± 2.72	85.61 ± 0.87	95.23 ± 0.90	78.15 ± 3.72
Cyanidin 3,5-*O*-diglucoside	72.52 ± 0.45	98.54 ± 0.48	99.54 ± 0.13	92.77 ± 0.48
Cyanidin 3-*O*-glucoside	67.52 ± 1.58	90.44 ± 0.81	98.88 ± 0.13	82.17 ± 0.08
Delphinidin 3-*O*-glucoside	69.95 ± 1.53	93.99 ± 3.40	98.94 ± 0.39	83.60 ± 0.46
Pelargolidin 3,5-*O*-diglucoside	84.11 ± 2.52	63.13 ± 0.23	80.42 ± 4.95	79.90 ± 0.81

## Abbreviations

ASE	Accelerated solvent extraction
COD	Chemical oxygen demand
HVED	High voltage electrical discharges
MAE	Microwave assisted extraction
MF	Microfiltration
MWCO	Molecular weight cut-off
NF	Nanofiltration
OMWs	Olive mill wastewaters
PA	Polyamide
PEF	Pulsed electric fields
PES	Polyethersulfone
POH	pulsed ohmic heating
PPs	Polyphenols
PS	Polysulfone
PVDF	Polyvinylidene fluoride
RO	Reverse osmosis
SbFE	Subcritical fluid extraction
SCFE	Supercritical fluid extraction
TAA	Total antioxidant activity
TMP	Transmembrane pressure
TOC	Total organic carbon
TSS	Total suspended solids
UAE	Ultrasound-assisted extraction
UF	Ultrafiltration
